# HLA-G: Too Much or Too Little? Role in Cancer and Autoimmune Disease

**DOI:** 10.3389/fimmu.2022.796054

**Published:** 2022-01-27

**Authors:** José Manuel Martín-Villa, Christian Vaquero-Yuste, Marta Molina-Alejandre, Ignacio Juarez, Fabio Suárez-Trujillo, Adrián López-Nares, José Palacio‐Gruber, Luis Barrera-Gutiérrez, Eduardo Fernández-Cruz, Carmen Rodríguez-Sainz, Antonio Arnaiz-Villena

**Affiliations:** ^1^Departamento de Inmunología, Oftalmología y ORL, Facultad de Medicina, Universidad Complutense de Madrid, Madrid, Spain; ^2^Instituto de Investigación Sanitaria Gregorio Marañón, Madrid, Spain; ^3^Servicio de Inmunología, Hospital Universitario Gregorio Marañón, Madrid, Spain

**Keywords:** HLA-G, Cancer, autoimmunity, immunoediting, checkpoint, ILT2, therapy, polymorphisms

## Abstract

HLA-G is a non-classical HLA class I molecule with immunomodulatory properties. It was initially described at the maternal-fetal interface, and it was later found that this molecule was constitutively expressed on certain immuneprivileged tissues, such as cornea, endothelial and erythroid precursors, and thymus. The immunosuppressive effect of HLA-G is exerted through the interaction with its cognate receptors, expressed on immunocompetent cells, like ILT2, expressed on NK, B, T cells and APCs; ILT4, on APCs; KIR, found on the surface of NK cells; and finally, the co-receptor CD8. Because of these immunomodulatory functions, HLA-G has been involved in several processes, amongst which organ transplantation, viral infections, cancer progression, and autoimmunity. HLA-G neo-expression on tumors has been recently described in several types of malignancies. In fact, tumor progression is tightly linked to the presence of the molecule, as it exerts its tolerogenic function, inhibiting the cells of the immune system and favoring tumor escape. Several polymorphisms in the 3’UTR region condition changes in HLA-G expression (14bp and +3142C/G, among others), which have been associated with both the development and outcome of patients with different tumor types. Also, in recent years, several studies have shown that HLA-G plays an important role in the control of autoimmune diseases. The ability of HLA-G to limit the progression of these diseases has been confirmed and, in fact, levels of the molecule and several of its polymorphisms have been associated with increased susceptibility to the development of autoimmune diseases, as well as increased disease severity. Thus, modulating HLA-G expression in target tissues of oncology patients or patients with autoimmune diseases may be potential therapeutic approaches to treat these pathological conditions.

## 1 HLA-G

HLA-G is a non-classical HLA class I gene that encodes a molecule with tolerogenic properties (1). This molecule shows restricted tissue expression pattern, and was initially observed in extravillous cytotrophoblasts, where it plays an important role in the maintenance of fetal-maternal immune tolerance (2); it has also been observed in few healthy immune-privileged tissues, as cornea (3) and thymic medulla (4, 5). However, HLA-G expression has also been reported in some pathological conditions, such as cancer and autoimmunity.

The *HLA-G* gene has a genetic structure similar to other classical HLA class I genes, although, in contrast, the sequence of the *HLA-G* gene is highly conserved (6).

**Figure 1:**
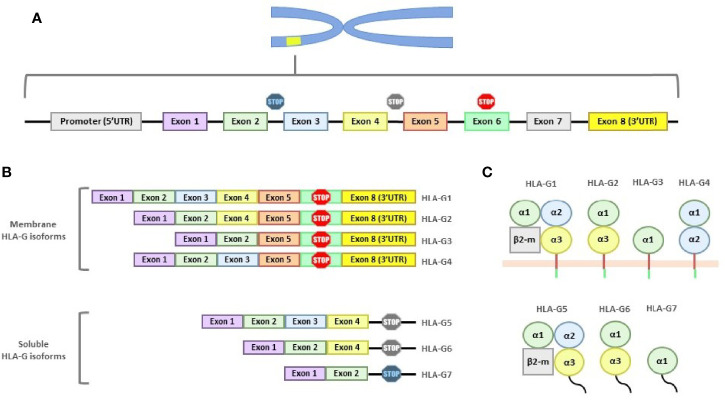
Structure of the HLA-G gene and its products. **(A)** HLA-G gene is composed of 7 introns and 8 exons. Exon 1 encodes the signal peptide; exons 2, 3 and 4 encode the α1, α2 and α3 subunits; exon 5 encodes the transmembrane region; and exon 6 the cytoplasmic region. Exons 7 and 8 are always transcribed, although exon 7 is never present in the mature mRNA and exon 8 (3’UTR region) is not translated due to the stop codon present in exon 6. **(B)** Mature mRNA of the different HLA-G isoforms. All isoforms show exon 1 and 2. Soluble isoforms are generated by alternative splicing which eliminates the exons involved in membrane binding. **(C)** The seven HLA-G isoforms, grouped whether they are membrane-bound or soluble, are shown.

The entire HLA-G molecule consists of a heavy chain, encoded on chromosome 6, non-covalently associated with β2 microglobulin, encoded on chromosome 15. Like classical HLA genes, the *HLA-G* gene has 7 introns and 8 exons. Exon 1 encodes the signal peptide, exons 2, 3 and 4 the extracellular domains α1, α2 and α3, respectively, and exons 5 and 6 the transmembrane and cytoplasmic domain, respectively (7) (**Figure 1A**).

Compared to classical class I molecules, HLA-G has a shortened cytoplasmic domain due to the presence of a premature stop codon in exon 6. Exon 7 is always absent from the mature mRNA and, as a consequence of this stop codon, exon 8 will never be present in the final protein. However, exon 8 forms the 3′ untranslated region (3’UTR), which is key in the regulation of *HLA-G* gene expression (8).

In total, seven isoforms of HLA-G mRNA generated by alternative splicing have been described, including four membrane-bound isoforms (HLA-G1, -G2, -G3 and -G4), and three soluble isoforms (HLA-G5, -G6 and -G7) (9) (**Figures 1B, C**).

## 2 *HLA-G* Gene Polymorphisms

The constitutive and inducible expression of HLA-G is primarily controlled by its promoter, which has unique structural characteristics such as enhancer A, interferon-stimulated regulatory element (ISRE) and SXY modulator, which contains regulatory sequences common to class I and II HLA genes (10, 11).

HLA-G expression may be controlled, as in any other gene, by regulatory regions both in the 5’URR and 3’UTR regions of the gene. The role of the 5’URR region in the expression of the HLA-G molecule and, thus, its involvement in pathology, is not as well known as that of the 3’UTR region, with scarce publications so far. Moreover, there are studies that question the impact of the 5’URR region on the expression of the HLA-G molecule and suggest that it alone cannot predict soluble HLA-G (sHLA-G) expression *in vivo* (12). This review will then focus on the 3’-UTR region of the *HLA-G* gene. HLA-G expression is highly regulated by its 3′UTR region, which has a high variability compared to the coding region, in contrast to classic class I HLA molecules (13).

**Figure 2:**
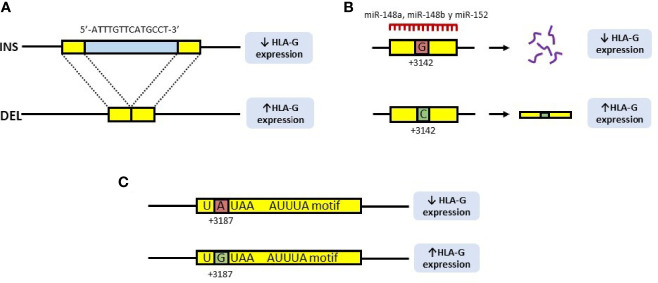
Polymorphisms of the 3’UTR region of the HLA-G gene involved in the expression of the HLA-G molecule. **(A)** 14bp INS/DEL polymorphism is characterized by the insertion (INS) or deletion (DEL) of a 14bp fragment at position +2960 of the *HLA-G* gene. Insertion is associated with decreased expression of HLA-G, due to a longer and unstable transcript, while deletion is associated with increased expression. **(B)** In the +3142C/G polymorphism, the presence of a guanine (G) at position +3142 of the *HLA-G* gene increases the affinity of the microRNAs, miR-148a, miR148b and miR-152 for this region, thus decreasing the availability of the mRNA. In contrast, the presence of a cytosine (C), produces an increase in the expression of HLA-G. **(C)** In the +3187A/G polymorphism, the presence of an adenine (A) at this position modifies an AU-rich motif in the gene mRNA, decreasing its stability, while G is associated with an increase in HLA-G production.

Population studies have found nine polymorphic sites in the 3’UTR region of the *HLA-G* gene. Among them, the 14 base pair (14bp) INS/DEL (rs371194629), +3142C/G (rs1063320) and +3187A/G (rs9380142) polymorphisms are implicated in HLA-G expression (14) (**Figure 2**).

Regarding the rs371194629 polymorphism, it consists of the presence (insertion, INS) or absence (deletion, DEL) of a 14bp fragment (7, 8). This fragment (5’-ATTTGTTCATGCCT-3’) is located at position +2960 of the 3’UTR region, and has been associated with both, the splicing and the stability of the mRNA (8, 15, 16), as it contains an AUUUG domain putatively exerting an AU-pentamer-like effect, decreasing mRNA stability (17). Therefore, the DEL allele provides a higher stability of the mRNA (15), associated with a high expression of HLA-G (16) (**Figure 2A**).

The rs1063320 polymorphism (+3142C/G), consists of the transversion of a cytosine (C) to guanine (G) at position +3142 of the 3’UTR region. The presence of a G increases the affinity of the microRNAs miR-148a, miR148b and miR-152 for this region, thus decreasing the availability of the mRNA by degradation of the primary transcript, as well as by the suppression of its translation (18). Should a C be found at this position, miRNAs affinity will decrease, increasing the mRNA availability and the production of HLA-G (8) (**Figure 2B**).

Finally, the +3187A/G polymorphism (rs9380142) is also implicated in the stability of HLA-G mRNA: the presence of an adenine (A) at this position modifies an AU-rich motif in the corresponding mRNA, decreasing its stability, while the G allele is associated with increased production of HLA-G (19) (**Figure 2C**).

## 3 HLA-G Function: Controlling the Immune Response

Several evidences have supported the role of HLA-G as a tolerance-inducing molecule, playing an important role in the suppression of the immune response. This molecule is able to exert this function by means of different strategies:

### 3.1 HLA-G Receptors

**Figure 3:**
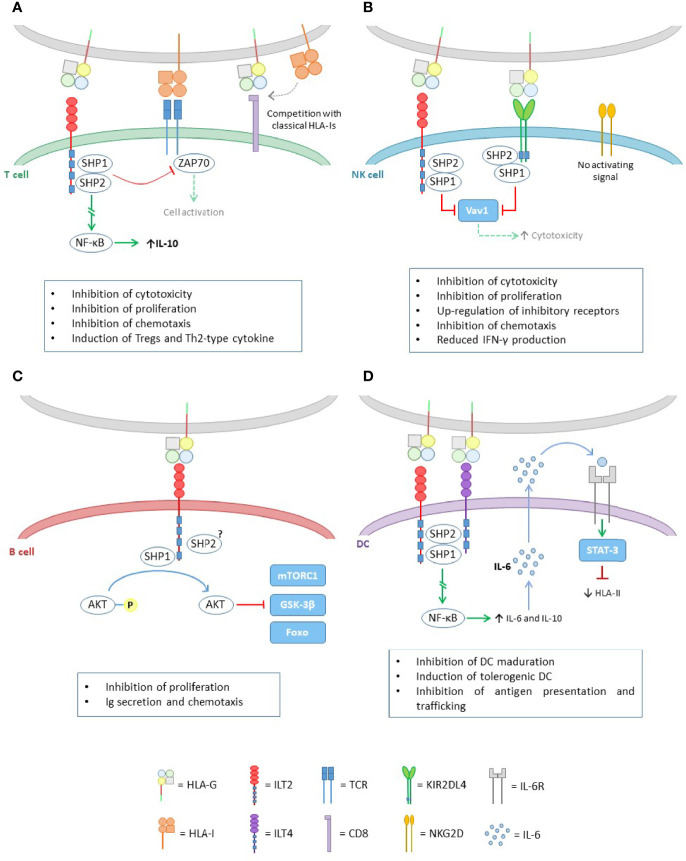
HLA-G effect in the different cells of the immune system. **(A)** Inhibitory effect of HLA-G on T cells. The binding of HLA-G to ILT2, activates the ITIM domains of the latter and recruits the SHP-1 and SHP-2 phosphatases. On the one hand, these proteins are capable of inhibiting the TCR signal, through ZAP70 dephosphorylation. On the other hand, they trigger a signaling pathway leading to NF-kB activation, which enhances IL-10 expression. HLA-G also exerts its inhibitory function through competition for the union of classical HLA class I molecules to the CD8 coreceptor in CD8+ T cells, and to the TCR. **(B)** HLA-G binds to its cognate receptors ILT2 and KIR2DL4, both containing inhibitory domains ITIM, which upon activation, recruits SHP-1 and SHP-2, leading to Vav1 inactivation, and thus downregulating the cytotoxicity capacity of NK cells. **(C)** In B cells, HLA-G works *via* ILT2, where SHP-1 mainly, is responsible for the dephosphorylation of AKT leading to mTORC1, GSK-3β and Foxo inactivation. **(D)** Like in T cells, the pathway initiated by HLA-G through ILT2 and ILT4 leads to NF-kB activation. This factor causes an increase in IL-6 and IL-10 expression. IL-6 is capable of binding to IL-6 receptors on DCs and, through the STAT-3 activation factor, inhibits HLA class II assembly.

The HLA-G molecule is able to bind to different inhibitory receptors present on cells of the immune system (**Figure 3**). The immunoglobulin-like transcription receptor type 2 (ILT2/LILRB1/CD85j) is expressed on T cells, B cells, natural killer (NK) cells and antigen presenting cells (APCs), whereas type 4 (ILT4/LILRB2/CD85d) is unique to APCs. Both ILT2 and ILT4 recognize HLA class I molecules; however, they bind with higher affinity (3- to 4-times fold) to the HLA-G molecule, which contributes mostly to the functional inhibition of cells expressing these receptors (20).

Another receptor that recognizes HLA-G is the killer cell immunoglobulin-like receptor (KIR) 2DL4 (CD158d), exclusive to NK cells. This receptor specifically recognizes HLA-G and has been associated with both activating and inhibitory functions. The mechanisms by which KIR2DL4 produces the activation of these cells are not clearly understood. However, it has been reported that it is limited to increasing interferon γ (IFNγ) production but not cytotoxic activity of non-activated NK cells (21). This increase in IFNγ would concomitantly lead to an increase in HLA-G expression (22).

All these receptors have in common that they display immunoreceptor tyrosine-based inhibition motifs (ITIMs) in their cytoplasmic tails, whereby HLA-G exerts its inhibitory functions. Upon HLA-G binding to its cognate receptors, the ITIM motifs recruits mainly the protein tyrosine phosphatase SHP-1, and also SHP-2 (20, 23), which dephosphorylate key points of the activating signaling pathways, causing distinct inhibitory effects on target cells.

In addition, the CD8 co-receptor is also able to recognize and bind to HLA-G. It has been described that the ILT2 receptor, expressed on CD8+ T lymphocytes, competes with this co-receptor for binding to HLA class I molecules, and therefore HLA-G is able to modulate the activation of cytotoxic T lymphocytes by blocking CD8 binding (20).

#### 3.1.1 T Cells

The binding of HLA-G to its receptors on T cells causes modifications in their function (**Figure 3A**). In general, they inhibit the proliferation of these cells (24) and also the cytotoxic function of CD8+ T cells (25), as well as the alloreactivity of CD4+ T cells (26). In addition, it has been reported that HLA-G can induce apoptosis of CD8+ cells (27, 28).

Also, the expression of HLA-G regulates the balance between T helper (Th) 1 and Th2 cells, promoting polarization to the Th2 subset. In fact, HLA-G decreases the production of IFNγ and tumor necrosis factor α (TNF-α) but increases the production of interleukin (IL)-3, IL-4 and IL-10 by Th2. IL-4 promotes differentiation to Th2 while IL-10 increases HLA-G expression in macrophages and monocytes activated by a feedback mechanism. Thus, HLA-G may have important implications in controlling the development of Th1- and Th2-mediated diseases (29).

#### 3.1.2 NK Cells

HLA-G exerts its effect on NK cells through ILT2 and KIR2DL4 (**Figure 3B**). Upon binding to these receptors, HLA-G is able to block certain NK signaling pathways, resulting in inhibition of the cytotoxic function (30) and transendothelial migration (31) of these cells. In addition, HLA-G promotes the up-regulation of the inhibitory receptors ILT2 and KIR2DL4 (32) and the secretion of pro-angiogenic factors, such as VEGF and angiopoietin-1 and -2, which allow early remodelling of the maternal vasculature during pregnancy, favoring the development of the fetus (33). This mechanism can be exploited by tumor cells, favoring tumor progression.

HLA-G can also enhance the surface expression of other non-classical HLA molecules, such as HLA-E, in target cells (34), which, through the CD94/NKG2A receptor, can also exert inhibitory effects on NK cells (35). Therefore, HLA-G allows an indirect inhibition of the NK cells cytotoxic function through stabilization of cell-surface HLA-E.

#### 3.1.3 B Cells

Like in T and NK cells, HLA-G exerts its immunosuppressive effect on B cells through the ILT2 receptor, leading to tolerance (**Figure 3C**).

HLA-G inhibits the response of both naive and memory B cells (36). It impairs cell proliferation and differentiation, as HLA-G causes key pathways for these processes to be inactivated and induces G0/G1 cell cycle arrest. Chemotaxis is also affected, as HLA-G causes a decrease in the expression of both CXCR4 and CXCR5, negatively influencing cell trafficking. Finally, the HLA-G/ILT2 pathway has been shown to inhibit antibody production induced by both T cell-dependent and -independent responses.

#### 3.1.4 Antigen Presenting Cells (APCs)

APCs are key cells in the activation of different immune cell types, influencing both innate and adaptive responses.

These cells have ILT-2/4 receptors which, when interacting with HLA-G, alters their behavior (**Figure 3D**). Generally, dendritic cells (DC) are the most commonly affected. HLA-G acts by inhibiting the differentiation and maturation of DCs, and also inducing the production of tolerogenic DCs.

The interaction with HLA-G also interferes with the assembly and transport of HLA class II molecules to the cell surface, decreasing the presentation of antigens to other cells of the immune system, thus disrupting the characteristic function of DCs (37).

Altogether, these induced tolerogenic DC are able to generate anergy in the CD4+ T population, in addition to favoring the appearance of CD4+CD25+CTLA-4+ and CD8+CD28- cells with a high capacity of IL-10 production, thus having suppressive and regulatory properties.

Because of the tolerogenic function of HLA-G in DCs, some works have tried to use these cells as a therapeutic approach to induce tolerance in some pathologies (38), such as multiple sclerosis (39).

Finally, as seen in the case of NK cells, HLA-G also induces up-regulation of inhibitory receptors on APCs (32).

### 3.2 Long-Term Tolerance Generation (Treg)

Regulatory T cells (Treg) are key cells in the maintenance of normal immune homeostasis. It has been observed that the HLA-G molecule, which is characterized by its tolerogenic function, promotes the differentiation of naïve T cells, both CD3+CD4low and CD3+CD8low, to suppressor T cells that, unlike natural regulatory T cells, do not express the Foxp3 transcription factor (40). This HLA-G-induced regulatory T-cell subset does not depend on HLA-G expression to exert its regulatory function. This suppressor T cells rely on the secretion of inhibitory cytokines, such as IL-10, which apart from inhibiting various immune cells populations, also up-regulates HLA-G expression (41) and enhances other cell-to-cell mechanisms that induce anergy in several cell types, such as CTLA-4/B7 (42), PDL1/PD1 (43), etc.

In addition, a population of regulatory T cells that constitutively expresses HLA-G has been described (44). These cells also mediate their immunosuppressive function through various tolerogenic cytokines such as IL-10, IL-35 and transforming growth factor (TGF)-β which, like IL-10, up-regulates HLA-G expression (45).

### 3.3 Short-Term Tolerance Generation

Trogocytosis is a process in which the rapid uptake of membranes and their associated molecules occurs through cell-to-cell contact. This phenomenon has been described in T and B lymphocytes, NK cells, APCs and even tumor cells (46, 47).

Some of these membrane-associated molecules are the various membrane-bound HLA-G isoforms. LeMaoult et al. (48) have found that, when contact occurs between an APC and a T lymphocyte, the T cell may acquire the HLA-G from the APC membrane and incorporates it into its membrane through this trogocytosis phenomenon. These HLA-G expressing T cells are then able to adopt a short-term regulatory function and, unlike Tregs, do not require specific cell maturation.

The same happens when contact occurs between a tumor cell expressing HLA-G on its membrane and a NK cell. When NK cells incorporate the membrane-bound isoforms of HLA-G, they stop proliferating, limit their cytotoxic activity and behave as suppressor cells. In addition, these cells can inhibit the cytotoxic function of other NK cells, contributing to tumor escape (49).

## 4 HLA-G and Pathology

As a consequence of this tolerogenic role, it has been postulated that HLA-G might be implicated in a wide variety of processes:

### 4.1 HLA-G and Transplant

The immunosuppressive role of HLA-G in transplants is directly related to graft survival, due to the induction of tolerance that this molecule exerts on the immune system (50). In transplanted patients, HLA-G expression may allow escape from recognition and destruction of the graft by the cytotoxic activity of T lymphocytes and NK cells (51), which are one of the main reasons for graft rejection (52), suggesting the potential role of HLA-G in the protection of the allograft.

The mechanism whereby HLA-G is expressed *de novo* in transplanted organs remains to be elucidated. Several factors, relevant in this context have been shown to induce *de novo* HLA-G expression *in vitro*, such as steroids used in acute rejection episodes, and cytokines expressed in allogeneic immune responses, like the pro-inflammatory cytokine IFNγ and the anti-inflammatory cytokine IL-10 (53).

Historically, the relationship between HLA-G and graft acceptance/rejection was first observed in heart transplantation (54), and, over time, information on this subject has been expanded. These studies reported the presence of HLA-G in biopsies of transplanted heart tissue, where HLA-G was especially prevalent in patients with no or low rejection scores (55). In HLA-G-positive patients, the incidence of acute or chronic rejection was significantly decreased (p<0.001 and p<0.032, respectively) when compared with HLA-G-negative patients (54).

Similar findings were reported in lung, liver and kidney transplant recipients (50, 55). Altogether, these results provided evidence of the immune inhibitory effect of HLA-G and pointed to its potential role as a biomarker of cellular rejection status.

As mentioned in the “HLA-G” section, this molecule presents both membrane and soluble isoforms. As membrane HLA-G and soluble HLA-G (sHLA-G) structures exhibit the same receptor specificity, both are potent molecules modulating the innate and adaptive immune response, with sHLA-G having the advantage of being free in the extracellular medium, being able to exert its effect in a paracrine or even endocrine fashion upon extravasation into the bloodstream.

This feature regarding sHLA-G is very important, since detection in blood is less invasive than detection of HLA-G in biopsies. In fact, some articles have seen that, after kidney transplantation, sHLA-G levels were detected in the blood of these patients, and patients with chronic rejection belong in the sHLA-G negative group (56). Thus, elevated levels of sHLA-G in blood of transplant patients are associated with better graft acceptance and a higher survival rate after transplantation (54).

### 4.2 HLA-G in Chronic Viral Infections

It is known that viral infections lead to a decrease in the expression of HLA class I molecules, including HLA-G, allowing NK cells to detect the infected cells and thus be able to perform their cytotoxic function on them. However, some viruses, such as herpes simplex virus type I (HSV-1) or the rabies virus (RABV), have developed resistance mechanisms based on the induction of the overexpression of HLA class I molecules, both classical and non-classical (such as HLA-G), which has an immunosuppressive effect on NK lymphocytes (57). Therefore, an increase in the HLA-G expression induced by the virus itself or by the inflammatory environment, can aggravate the morbidity or mortality of the infection (58, 59).

### 4.3 HLA-G and Cancer

Tumor cells present, anchored to MHC class I molecules, certain tumor-derived antigens on their surfaces that can be recognized by the patient’s immune system. However, even in an immunocompetent organism, neoplastic cells are able to grow and progress, leading to aggressive and malignant lesions. In this context, immunoregulatory molecules, like HLA-G, play an important role in the progression of cancer.

In addition to its localization and physiological function, HLA-G expression has been observed in different types of tumors, such as gastric (60), colorectal (61) and breast (62) cancer, among others, where it favors tumor progression by inhibiting the immune system surveillance (63).

### 4.4 HLA-G and Autoimmunity

Autoimmune diseases comprise a very heterogeneous group of pathologies, whose main feature is the exacerbation of the immune response against self-antigens of the organism.

Therefore, the presence of tolerogenic mechanisms is fundamental, such as the AIRE transcription factor, which allows negative selection of developing thymocytes. According to this, Melo-Lima et al. (5) have been able to prove that this factor up-regulates HLA-G expression in thymic cells, limiting autoimmune diseases (59).

Due to the relevant implications of HLA-G in cancer and autoimmunity, we will describe in detail the most relevant studies of HLA-G in different tumor types and autoimmune diseases in the next sections of this review.

## 5 Cancer

Tumors are complex tissues composed not only of tumor cells, but also a repertoire of immune cells (microenvironment) in a continuous crosstalk with the malignant cells. The immune microenvironment can recognize tumor cells as “foreign” and initiate mechanisms to eliminate tumor cells, while malignant cells are able to prevent the action of the immune cells, by releasing immune suppressing extracellular signals and taking advantage of several molecular mechanisms to frustrate immune-mediated death.

Thus, we know as “immunosurveillance” the extrinsic mechanism of cancer suppression that eliminates emerging tumors. Therefore, escape from this immunosurveillance represents an essential step in the development of neoplastic diseases.

One of the molecules that tumors use to their advantage for this purpose is HLA-G which, as mentioned previously, has tolerogenic capabilities that could benefit tumors. In fact, over the years, many studies have reported on the expression of HLA-G in many different types of cancers, which supports the idea that this molecule participates in cancer development.

**Figure 4:**
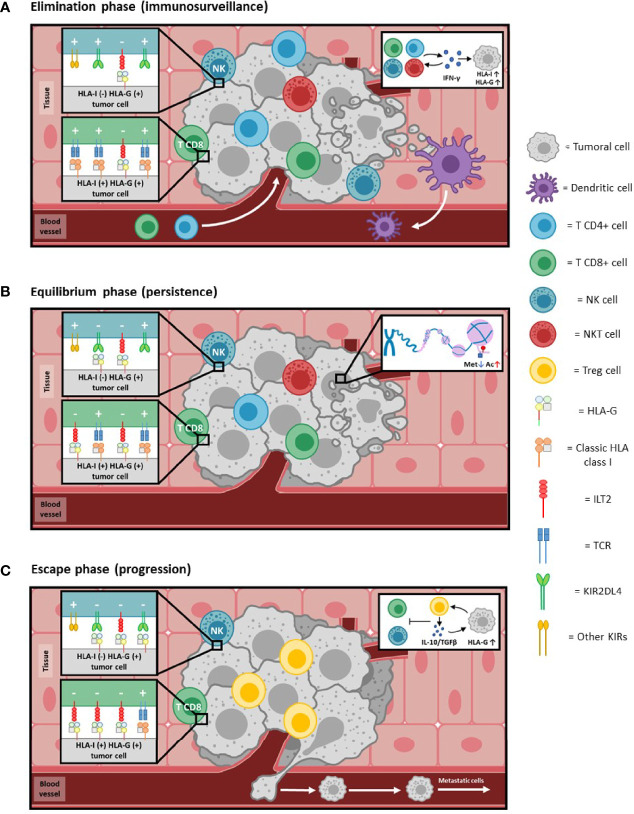
HLA-G and cancer immunoediting. Immunoediting of cancer, a phenomenon whereby cancer and immunocompetent cells interplay, leading in some instances to tumor overgrowth. Consists of three phases (see references 63-66 for details): **(A)** Elimination phase: In reply to the neoplastic process, immunocompetent cells extravasate and infiltrate the tumoral tissue. The combined action of both innate and adaptive immunity effectors detects and kills malignant cells. The production of cytokines, such as IFNγ (see insert 4A), induces an increase in the expression of HLA class I molecules. Cells that present tumor antigens through HLA class I molecules [HLA-I (+)] are targeted mainly by CD8+ T lymphocytes. Those that do not present class I molecules on their surface [HLA-I (-)] are eliminated by NK cells. The expression of HLA-G can also be induced [HLA-G (+)] by IFNγ, although to such low levels compared to HLA class I that its inhibitory activity (-) is negligible compared to the activating (+) capacity, and thus the tumor is controlled by the immune system. **(B)** Equilibrium phase: As adaptive immunity continues to eliminate tumor cells, it simultaneously exerts selective pressure leading to the generation of less immunogenic malignant cells (e.g., selective or complete loss of HLA class I molecules and/or tumor antigens). Genetic instability resulting from defective intracellular control mechanisms, as well as frequent epigenetic changes, further contribute to the development of a non-immunogenic phenotype. Histone demethylation and acetylation processes (insert 4B) lead to epigenetic activation of HLA-G, allowing its expression to increase to levels comparable to the HLA class I molecules present at that time. Activation (+) and inhibition (-) signals will be balanced, leading to an equilibrium process in which the immune system becomes progressively less able to eliminate the tumor. **(C)** Escape phase: Tumor cells that have lost molecules relevant for immune recognition [HLA-I (-)] now tend to express higher levels of HLA-G on their surface [HLA-G (+)]. HLA-G creates peripheral tolerance by inducing anergy, immunosuppressive cells (Treg) and production of immunosuppressive cytokines (IL-10 and TGFβ; insert 4C). In addition to the local effects of membrane-bound HLA-G, secreted soluble HLA-G could also have systemic inhibitory activity due to its distribution through the blood circulation. Inhibiting signals (-) overcome activating (+) ones in immunocompetent cells. Taken together, this allows complete escape by the tumor from the action of the immune system, which favors its development and processes such as metastasis.

Thanks to the ability of tumors to escape from the immune system, the mechanism of “cancer immunoediting” arises, by which immunoresistant tumor variants are generated and in which molecules such as HLA-G participate (63–66) (**Figure 4**). This process is divided into three phases:

- Elimination: It corresponds to the immunosurveillance mechanism, and involves the elimination of tumor cells by cells of innate (NK, NKT, LTγδ, DC, etc) and adaptive (antigen-specific LT and LB) immunity. In this phase, the activity of tumor infiltrating lymphocytes (TILs) and NK cells is associated with cytotoxicity and with the production of Th1 profile cytokines (IFNγ), that increase the cell surface expression of HLA class I molecules, including HLA-G (**Figure 4A**).
- Equilibrium: The immune system exerts a selective pressure on tumor cells which, together with genetic instability and frequent epigenetic changes, leads to the generation of clones with less immunogenicity, resistant to the action of the immune system. An example of this process is the selective loss of classical HLA class I molecules, which prevents the presentation of tumor antigens and thus avoids the attack by adaptive immunity.

Also, during this phase, epigenetic mechanisms lead to a process of histone hypomethylation and acetylation that favors the expression of HLA-G, which competes with the antigenic presentation by classical histocompatibility molecules, promoting a process of tumor tolerance (**Figure 4B**).

- Escape: In this phase, high increase of HLA-G expression, together with other mechanisms takes place; this allows complete evasion of the immune system by the tumor. HLA-G induces anergy and apoptosis of immune effector cells, and promotes the production of Treg cells and immunosuppressive cytokines (IL-10).

With this, malignant cells are able to evade the immune response, extravasate into the circulation and generate long-distance metastases (**Figure 4C**).

The presence of HLA-G in tumors has been described in several studies, some of them emphasizing that it is only present in tumor and not in distal/healthy tissue (60, 61, 67, 68), a finding that would reinforce the idea of the participation of HLA-G in tumor development and progression.

To describe the effect of HLA-G in the context of tumor progression, we refer here some of the most studied types of cancer:

### 5.1 Gastric Cancer

As previously mentioned, HLA-G expression is highly dependent on different polymorphisms, especially those present in the 3’UTR region, which affect the stability of HLA-G mRNA. For this reason, analyzing the genetic association of HLA-G polymorphic variants and the development or progression of pathologies dependent on HLA-G inhibitory activity could establish HLA-G polymorphism as a risk factor for several diseases.

In fact, in a study performed by our group in a cohort of 107 patients with gastric cancer and 58 controls in which we analyzed the 14bp INS/DEL and +3142C/G polymorphisms (69), the implication of the DEL allele in gastric cancer (70.0% in patients vs 57.0% in controls, p=0.025) was assessed, as well as that of the haplotype formed by the combination of the 14bp DEL/+3142C variants, also increased in patients compared to controls (54.1% vs 44.4%, p=0.034).

In addition, Kaplan-Meier analysis revealed that 14bp DEL/DEL patients showed lower 5-year life-expectancy than INS/DEL or INS/INS (p=0.041). Therefore, we concluded that the 14bp INS/DEL and +3142C/G polymorphisms of the *HLA-G* gene mediate gastric cancer risk and survival, becoming important risk factors to be taken into account.

Although the effect of these polymorphisms on the mRNA and protein expression are well established, a limitation for the majority of this kind of studies is that the correlation between polymorphisms and HLA-G expression is not analyzed. In our case, we have detected HLA-G in tissue (immunohistochemistry) and sHLA-G in plasma (ELISA) (69), although that correlation was not explored.

As mentioned, in addition to genetic studies, determination of HLA-G expression is key to understanding the involvement of this molecule in cancer. Wan et al. (60) observed both HLA-G expression in 61.2% of their patients and that it correlated with tumor invasion depth (p=0.012), lymph node metastasis (p=0.015) and clinical stages (p=0.001). In addition, patients with HLA-G-positive (+) tumors had a worse prognosis than HLA-G-negative (–) patients. Importantly, they found that the number of tumor-infiltrating NK cells was significantly lower in HLA-G (+) tumors compared to HLA-G (–) tumors (p<0.001), emphasizing the inhibitory role of this molecule.

The study by Murdaca et al. (70) reinforces what was observed for gastric cancer. The presence of HLA-G in tumor tissue (25.5% of patients) was also reported, and the presence of HLA-G correlated with worse patient survival (p<0.0001).

### 5.2 Colorectal Cancer

In addition to analyzing the three most classic polymorphisms of the *HLA-G* gene (14bp INS/DEL, +3142C/G and +3187A/G), Garziera et al. (71) analyzed the +3035 C/T polymorphism, among others, which also affect HLA-G expression. As for the 14bp INS/DEL polymorphism, carriers of the DEL/DEL allele (higher HLA-G expression) had lower disease-free survival rate, lower overall survival rate and higher risk of recurrence compared to the other genotypes.

Similar results were found for the +3187G/G genotype, which increases the HLA-G mRNA stability and, therefore, HLA-G molecule expression, thus favoring tumor progression, as expected. A similar finding was also observed for the +3035C/C genotype of the +3035T/C polymorphism. Altogether, authors concluded that +3035C>T and, in particular, +2960 14bp INS/DEL and +3187A/G polymorphisms were prognostic biomarkers in determining survival outcome in colorectal cancer (CRC) (71).

The study by Ye et al. (61), in addition to proving the expression of HLA-G in 64.6% of CRC patients, allowed to establish a relationship between HLA-G expression and the risk of developing this disease. A statistically significant correlation was found between HLA-G protein levels and clinicopathological factors of depth of invasion, histological grade, host immune response, nodal status and clinical stages of the disease. They also found that the presence of HLA-G negatively affected in a significant manner the 3-year survival of patients (96.0% in HLA-G (-) vs 51.0% in HLA-G (+), p=0.001).

Interestingly, Lázaro-Sánchez et al. (72) found that patients with CRC have significantly higher sHLA-G levels in saliva than control subjects (18.8 U/ml vs 6.3 U/ml, p = 0.036). In addition, higher levels of sHLA-G were observed in the saliva of patients with CRC in more advanced stages, compared with patients in early stages (24.2 U/ml vs. 8.1 U/ml, p = 0.019). Therefore, sHLA-G is a potential new biomarker for this type of cancer, which, furthermore, can be detected in a non-invasive way.

### 5.3 Breast Cancer

Regarding breast cancer, Eskandari-Nasab et al. (73) found a statistically significant difference in the frequency of the DEL allele in breast cancer patients compared to the control group (56.4% vs 46.5%, respectively. p=0.004). Likewise, the prevalence of the HLA-G 14bp DEL/DEL genotype was higher in breast cancer patients than in the control group (33.9% vs 24.1%, respectively, p=0.006), suggesting that the 14bp INS/DEL polymorphism could be a genetic risk factor mediating susceptibility to breast carcinoma.

As for the +3142C/G polymorphism, Zidi et al. (74) observed that both the G allele and the G/G genotype have a protective effect for breast cancer risk (p=0.0004 and p=0.0005, respectively), as they are less prevalent in patients compared to controls. This is consistent with the fact that the +3142G variant implies lower mRNA stability and lower HLA-G production, and therefore lower tolerogenic capacity in carriers.

An interesting research was carried out by He et al. (62). They found HLA-G expression in 66.0% of their patients. This expression significantly correlated with tumor size, nodal status, and clinical disease stage (p=0.0001, 0.012, and 0.0001, respectively). In addition, sHLA-G levels were higher in patients compared to controls (p<0.001). HLA-G expression also correlated with survival, as the patients with higher expression had a worse outcome (p=0.028). They also correlated HLA-G expression with the host immune response (measured as the number of tumor infiltrating lymphocytes), finding that the higher the HLA-G expression the less lymphocytes (p=0.011).

Altogether, these results suggest that HLA-G may have potential clinical implications in diagnosis and prognosis of patients with breast cancer.

### 5.4 Esophageal Cancer

Chen et al. (75) found that individuals carrying the 14bp DEL/DEL genotype had a 2.69-fold increased risk of suffering esophageal cancer compared with those carrying the 14bp INS/INS genotype (p=0.04). Further, individuals carrying the 14bp DEL/DEL and +3142C/C genotypes (DEL/C haplotype, which combines HLA-G-enhancing variants) had a 2.82-fold increased risk of esophageal cancer compared with individuals carrying the INS/C haplotype (p=0.04).

Regarding HLA-G expression in this type of cancer, Zheng et al. (68) observed that in 70.0% of the patients analyzed a positive HLA-G staining was achieved (p<0.05), and this correlated with cancer cell differentiation (p=0.033) and lymph node metastasis (p=0.035). A significant difference in plasma sHLA-G levels was also found between patients (15.04 U/mL) and healthy controls (6.81 U/mL, p<0.01). In addition, HLA-G-positive patients showed a poor prognosis. Interestingly, IL-10, an inhibitory cytokine whose expression is enhanced by HLA-G, has its levels increased in patients compared to controls (23.86 pg/mL vs. 12.81 pg/mL, p<0.01).

Hence, HLA-G is a potential predictive biomarker of esophageal cancer, and the modification of HLA-G transcription or expression may be of benefit in the prevention and treatment of this type of cancer.

### 5.5 Lung Cancer

There is also evidence of a link between HLA-G and lung cancer. Yie et al. (76) analyzed a cohort of 106 patients, finding HLA-G overexpression in 75.0% of cases. This expression significantly correlated with lymph nodal metastasis and clinical stages of the disease (p=0.0001 in all instances), and they observed lower presence of infiltrating lymphocytes in the areas where HLA-G was abundant compared to regions with scarce HLA-G (p=0.027), thus emphasizing the inhibitory role of HLA-G. Again, the overall survival rate of patients with HLA-G (-) tumors was significantly higher when compared to those with HLA-G (+) tumors (50.0% vs 22.0%; p=0.001).

HLA-G has also been correlated with worse prognosis and survival in other malignances such as hepatocellular cancer (77, 78), renal cancer (79) and pancreatic cancer (80).

### 5.6 Limitation of HLA-G Expression Analysis in Tissues

It is now widely accepted that HLA-G is a critical marker of immunotolerance in cancer immune evasion and is strongly associated with disease progress and prognosis in cancer patients. However, not all published works are equally consistent and, although they show HLA-G expression in cancer, do not allow to correlate HLA-G with certain types of cancer (81).

The reason for this discrepancy lies mainly in two points: inter- and intra-tumor heterogeneity and the different methodological approaches used for detecting HLA-G.

Regarding heterogeneity, multiple transcriptional, epigenetic, post-transcriptional and environmental mechanisms are involved in modulating protein expression in cancer. This also affects HLA-G, in addition to its genetic background (polymorphisms), which leads to the discrepancies mentioned earlier (82, 83).

The other problem lies in the methodological approaches employed. The vast majority of HLA-G detection studies use immunohistochemistry as the main method, but each laboratory uses different antibodies and variable experimental conditions (incubation times, antibody concentration, etc), even when analyzing the same type of tumor (81–83). In addition, some of these antibodies used, such as the 4H84 clone, appear to be poorly specific for HLA-G detection (84). This complicates the ability to interpret and compare different studies. It would be advisable to establish common experimental procedures to assess which of the available antibodies should be used in all the studies.

## 6 Autoimmunity

In recent years, several studies have shown that HLA-G plays an important role in the control of autoimmune and inflammatory diseases, caused by the uncontrolled activation of immune system cells (85). The ability of HLA-G to limit the progression of these diseases has been confirmed and, in fact, the levels of the molecule or the distribution of the polymorphisms of the gene modulating them, have been associated with an increased susceptibility to the development and severity of these diseases (86).

### 6.1 Systemic Lupus Erythematosus

Systemic Lupus Erythematosus (SLE) is a chronic autoimmune inflammatory disease that involves many organs and systems. It has been described that patients with this disease show a significant increase in the frequency of the 14bp INS/INS genotype of the 14bp INS/DEL polymorphism, compared to healthy controls (87). In addition, patients with SLE have a significant increase in the +3142G allele and the +3142G/G genotype of the +3142C>G polymorphism, associated with a lower expression of HLA-G due to increased degradation of the primary transcript, as well as by suppression of its translation (18, 88). In fact, a significant decrease of sHLA-G has been found in the plasma of these patients compared to healthy controls, which is usually associated with the 14bp INS/INS genotype (17, 89).

### 6.2 Multiple Sclerosis

Another disease in which the HLA-G molecule is implicated is Multiple Sclerosis (MS), the most common disabling neurological disorder in young adults (90). Studies to date have revealed that MS patients have a significantly higher frequency of the 14bp INS allele of the 14bp INS/DEL polymorphism than healthy controls and, in addition, the 14bp INS/INS genotype has been correlated with decreased plasma sHLA-G in these patients (91). On the other hand, an increase in the frequency of the +3142G allele of the +3142C>G polymorphism has also been observed in these patients compared to healthy controls and, in fact, the +3142C/C genotype is considered protective for this disease, appearing more frequently in the group of healthy controls (92).

### 6.3 Rheumatoid Arthritis

The HLA-G molecule is also one of the factors that contribute to the development of Rheumatoid Arthritis (RA), which involves chronic inflammation of the synovial membrane (93). A case-control study revealed that RA patients had a significant increase in the +3142G/G genotype of the +3142C/G polymorphism. In addition, patients with this genotype had significantly higher plasma sHLA-G levels than healthy controls (94). However, in contrast to other autoimmune diseases, there is some controversy in the association of the 14bp INS/DEL polymorphism and the risk or protection of this disease. Nevertheless, the possibility that RA is associated with this polymorphism cannot be ruled out due to lack of statistical power, considering that sample size is a critical factor in this type of study (95). Further research in this pathology is needed to unveil the implication of HLA-G in the development or severity of RA.

### 6.4 Type 1 Diabetes Mellitus

Some of the *HLA-G* gene polymorphisms and molecule levels have also been correlated with Type 1 Diabetes mellitus (T1D), a chronic and genetically complex disease characterized by pancreatic beta-cell destruction, mediated by humoral and cellular immune responses. The 14bp INS allele of the 14bp INS/DEL polymorphism appears significantly more frequent in patients than in healthy controls (96). Considering that the HLA-G molecule is constitutively expressed in the pancreas, where it protects the organ against cytotoxic cells, decreased expression level should be detrimental in individuals genetically prone to producing less HLA-G (97).

### 6.5 Psoriasis

In the case of psoriasis, a chronic inflammation in which environmental and genetic factors are involved, patients with the 14bp DEL allele and the DEL/DEL genotype of the 14bp INS/DEL polymorphism respond better to treatment with acitretin (98); so the analysis of this polymorphism could contribute to the development of personalized treatments for these patients.

### 6.6 Uveitis

Uveitis is a disorder characterized by inflammation of the uvea. Regarding this pathology, Crabtree et al. (99) have described that an increase of HLA-G induced in an animal model of experimental autoimmune uveitis (both soluble and membrane-bound), reduces the severity of the pathology in these mice, thus reiterating the benefit of the tolerogenic role of the HLA-G molecule in autoimmune/inflammatory diseases.

Uveitis is one of the possible clinical manifestations in patients suffering from Behçet’s syndrome, a systemic condition. Patients suffering from this syndrome show a significant increase in the frequency of the INS 14bp allele compared to controls and, in fact, patients with homozygous INS/INS genotype have twice the risk of suffering from this disease compared to healthy controls (100).

Furthermore, Park et al. (101) confirmed that the HLA-G*01:01:02 allele significantly increases the risk of the Behçet disease, since it contains the insertion of the 14bp fragment of 14bp INS/DEL polymorphism in 3’UTR region, which is associated with lower HLA-G expression. Also, the G*01:05N allele, that is a non-functional allele and does not encode either soluble isoforms or the membrane-bound G1 isoform, is significantly more frequent in patients compared to controls, thus being associated with higher risk to develop Behçet disease.

## 7 HLA-G and Therapy

### 7.1 Cancer

As shown in the present review, HLA-G expression has been reported in a wide variety of tumors, correlating significantly with the clinical outcome of patients. Both genetic (polymorphisms) and protein expression studies confirm that HLA-G overexpression in cancer is related to faster disease progression and worse clinical outcome. In fact, some of these studies (60, 62), clearly show a decrease in immunocompetent cells in an HLA-G rich tumor microenvironment, emphasizing the tolerogenic role of this molecule. Therefore, HLA-G and the downstream signaling pathways upon interaction with its cognate receptors can be soundly considered a new target for immune-based anti-tumor therapy.

One approach for anti-HLA-G therapy is downregulating HLA-G expression with miRNAs. This has been proposed by Kaminski et al. (102) in a clinical setting different from cancer (pregnancy). They propose to deliver miR-148a and miR-152 microRNAs, carried by liposomes, into the target cells, where they would interfere with the production of HLA-G (as mentioned in the “*HLA-G* gene polymorphisms” section).

Fu et al. (103) found that HLA-G expression was directly regulated by miR-152, and also that an aberrant expression of miR-152 influenced tolerance to, in this case, NK cell cytolysis *in vitro*. The cell lysis rate increased significantly when overexpressing miR-152 in the target cells (A549 cells), because, as a consequence of this, they had less HLA-G to protect themselves from NK cell-mediated lysis. They also did *in vivo* studies where they inoculated HLA-G expressing or HLA-G devoid (transfected with si-HLA-G) A549 cells in mice, finding that, in the latter case, the size of the tumors generated was significantly smaller than in the former. Thus, HLA-G expression was correlated with the growth and immune escape ability of tumoral (A549) cells *in vivo* and *in vitro*.

Based on the two aforementioned works (102, 103), it seems interesting to develop therapies with miRNAs aimed at reducing HLA-G expression in cancer, in order to restore the anti-tumor activity of cells restrained by this molecule.

On the other hand, the immune checkpoint inhibitor (ICI) therapy merits further attention. This type of therapy has been a major breakthrough in cancer treatment. However, despite the good achievements of therapeutic approaches such as anti PD1/PDL1, they have limitations, and there are still patients who do not benefit from this type of treatments (104). This is why there is a need to continue searching for new alternatives.

A recent study by Dumont et al. (105) lays the foundations to consider the HLA-G/ILT2 pathway as a new checkpoint. They characterized the CD8+ILT2+ lymphocyte population as antigen-experienced cells and highly cytotoxic, more so than CD8+PD1+ (the main target of ICI).

CD8+ILT2+ cells markedly upregulated cytotoxicity-related genes, consistent with the expression of KLRG1, perforin, and granzyme B, observed by flow cytometry. When CD3 activation assays were performed, in comparison with CD8+ILT2- and CD8+PD1+ cells, CD8+ILT2+ cells exhibited the highest degranulation rate (measured by CD107a and IFNγ). Therefore, these cells exhibit a high anti-tumor capacity.

Importantly, they assessed that these CD8+ILT2+ cells were specifically inhibited by the presence of HLA-G. When cocultured with HLA-G-expressing cells, a reduction in degranulation and IFNγ production was observed, thus reducing the anti-tumoral capacity of these cells. However, when using antibodies that interfere with the interaction between HLA-G and ILT2, CD8+ILT2+ cell activity was completely restored, blocking the immunosuppressive capacity of HLA-G. Accordingly, as with PD1/PDL1, an anti-HLA-G/ILT2 therapy could be effective for cancer treatment.

Additionally, Fu et al. (103) performed blockade studies of the HLA-G/ILT2 pathway in NK cells, also finding *in vitro* that using anti-ILT2 antibodies restored the cytotoxic capacity of these cells.

Also, and similar to the experiments mentioned above, *in vivo* studies were performed by transfecting mice with A549 cells and injecting them in the presence or absence of anti-ILT2 antibodies. Although no significant differences were clearly observed, tumors of the mice treated with anti-ILT2 were, nevertheless, smaller than in untreated mice, suggesting a blocking effect on the anti-tumor capacity of HLA-G. Consequently, blocking ILT2 maintained the lytic effect of NK cells in the presence of HLA-G, making the use of this anti-ILT2 a promising therapy for cancer.

Because PD1/PDL1 and HLA-G/ILT2 seem to work as independent mechanisms, a combination of therapies would cover a wider range of cells to restore the anti-tumor activity of the immune system.

Still, further investigation is needed to establish whether targeting HLA-G and its receptors is truly a robust therapy. More studies, such as those of Dumont et al. (105) and Fu et al. (103) are needed to fully elucidate the relevance of the HLA-G/ILT2 pathway, along with all other HLA-G mediated signaling pathways, involving pertinent immune cells.

### 7.2 Autoimmune Diseases

Currently, no HLA-G-based therapies have been developed for the treatment of autoimmune and autoinflammatory diseases. Despite the lack of studies in patients, a study performed in a murine model of experimental uveitis found that increasing HLA-G levels in these mice, significantly improved the clinical manifestations of this pathology, which confers this molecule a therapeutic potential for patients suffering from this type of diseases (99).

In addition, some of the HLA-G gene polymorphisms have been associated with response to certain autoinflammatory disease treatments. For example, the DEL/DEL genotype of the 14bp INS/DEL polymorphism has been associated with a better response to treatment with methotrexate in patients with rheumatologic diseases (59).

## 8 Conclusion

Given the tolerogenic function of HLA-G, this molecule plays a key role in modulating the immune response in several pathologies. Measuring the protein levels (whether membrane bound or soluble) or analyzing the gene polymorphisms involved in its expression, is of interest in cancer susceptibility and progression and in autoimmune diseases.

To the best of our knowledge, no HLA-G variants are currently used as diagnostic markers in current clinical settings. While no HLA-G allele can be used as a clear susceptibility marker, it holds true that some markers identify early on cancer patients who might present with worse clinical evolution and shorter life expectancy of the disease, as has been reported in gastric adenocarcinoma (69), colorectal cancer (61, 71) and breast cancer (73), among others.

In depth knowledge of the function of HLA-G opens the possibility of establishing new immunomodulatory therapeutic approaches in cancer (new immune checkpoint inhibitors) or in autoimmune diseases (modulating the levels of this molecule).

## Author Contributions

JMM-V: Literature research, writing and revision. CV-Y and MM-A: Experimental work, literature research, writing and figure drawing. IJ: Experimental work, literature research, writing and revision. FS-T, AL-N, JP-G, LB-G, EF-C, and CR-S: writing and revision. AA-V: Literature research, writing and revision.

## Funding

This work was supported by grants from Instituto de Salud Carlos III (PI18/00626, PI18/00721 and PI18-01695), with funds from the European Union (Fondo Europeo de Desarrollo Regional FEDER). IJ is a grant recipient of a Universidad Complutense de Madrid—Real Colegio Complutense Harvard grant (Ayudas para contratos predoctorales de personal investigador en formación CT18/16).

## Conflict of Interest

The authors declare that the research was conducted in the absence of any commercial or financial relationships that could be construed as a potential conflict of interest.

## Publisher’s Note

All claims expressed in this article are solely those of the authors and do not necessarily represent those of their affiliated organizations, or those of the publisher, the editors and the reviewers. Any product that may be evaluated in this article, or claim that may be made by its manufacturer, is not guaranteed or endorsed by the publisher.
